# The causal relationship between thoracic aortic aneurysm and immune cells: a mendelian randomization study

**DOI:** 10.1186/s12872-024-03876-1

**Published:** 2024-04-16

**Authors:** Guoli Liu, Sha Pan, Hongli Xia, Mincai Li, Ansen Wu

**Affiliations:** 1 Deparment of Vascular surgery, Xianning Central Hospital, the First Affiliated Hospital of Hubei University of Science and Technology, Xianning, Hubei P. R. China; 2 Department of Neonatology, Xianning Central Hospital, the First Affiliated Hospital of Hubei University of Science and Technology, Xianning, Hubei P. R. China; 3 Hubei University of Science and Technology, Xianning, 437100 Hubei P. R. China

**Keywords:** Mendelian randomization, Thoracic aortic aneurysm, Genome-wide association study, Immune cells, The causal link

## Abstract

One of the pathogenic causes of thoracic aortic aneurysm (TAA), a dangerous vascular condition that can cause aortic rupture, is autoimmune disorders. Currently, immune cell clustering is becoming more and more refined, and the specific immune cell phenotypes involved are yet unknown. Here, we want to clarify the causal link between TAA risk and 731 immune cell traits. There was a Mendelian randomization analysis (MR). We discovered that the presence of TAA led to an increase in CD45 on CD33^−^ HLA-DR^−^ myeloid cells, an increase in CD45 on natural killer cells, and a decrease in FSC-A on granulocytes after applying FDR correction. Our research also revealed a strong correlation between the incidence of TAA and an increase in immune cells with CD3 on CD39^+^ CD4^+^, and CD25 on IgD^−^ CD27^−^ phenotypes. Through genetic techniques, our research has shown the intimate relationship between immune cells and TAA, offering direction for future clinical investigations.

**Supplementary Information:**

The online version contains supplementary material available at 10.1186/s12872-024-03876-1.

## Introduction

A thoracic aortic aneurysm (TAA) is characterized by abnormal enlargement, distortion, and a tumor-like protrusion in a segment of the thoracic aorta. The most severe manifestation of TAA is aortic dissection or rupture, yet a significant number of patients remain asymptomatic [1]. The recognized causes include syphilis infection, autoimmune diseases (such as arthritis), connective tissue disorders (such as Marfan syndrome, Loeys Dietz syndrome, and Ehlers-Danlos syndrome), vascular degeneration (linked to factors like smoking and hypertension), and the presence of a bicuspid aortic valve [2]. At present, antihypertensive medications stand as the sole effective palliative treatment for TAA, while surgical replacement of the dilated aorta remains the only proven treatment strategy [3].

Evidence indicates an overexpression of immune response genes in the aortic media of dilated TAA samples, suggesting that inflammation plays a more significant role in the development of TAA in these patients [4, 5]. The role of immune cells in inflammation is substantial; thus, there is a close relationship between immune cells and the onset and progression of TAA [6]. In recent years, there has been a growing identification of new immune cell types with unique immunological roles. A comprehensive investigation into the relationship between TAA and immune cells is imperative. Determining the precise causative association between immune cells and TAA in traditional observational research is challenging due to limitations in sample size, potential reverse causal bias, and the presence of confounding variables [7].

To assess the causal relationship between immune cells and TAA, large-scale whole-genome association studies (GWAS) and Mendelian randomization (MR) techniques can be employed. These approaches help reverse causal correlations in causal inference and mitigate the impact of confounding variables [8]. GWAS serves as a powerful and reliable tool for MR research. Therefore, we gathered GWAS data encompassing 731 distinct types of immune cells and TAA. Subsequently, we utilized MR analysis to delve into the causal relationship between immune cells and TAA.

## Method

### Study design

Through a two-sample MR analysis, we systematically examined the causal relationship between 731 distinct types of immune cells and TAA. In this statistical method, three key hypotheses were employed to infer the causal connection between exposure factors and outcomes: (1) instrumental variables are strongly associated with exposure factors, (2) instrumental variables are not associated with confounding factors, and (3) instrumental variables are only associated with exposure and outcomes.

### Data sources for TAA and 731 immune cells

The Genome-Wide Association Study (GWAS) Catalog offers summary statistics for TAA (GCST90027266) and 731 immune cell traits (accession numbers GCST0002121 to GCST0001391) from publicly accessible GWAS datasets. The TAA data, based on European ethnicity, comprises 1,351 cases and 18,295 controls. Following quality control and attribution, an examination of more than 23 million single nucleotide polymorphisms (SNPs) was conducted [9]. As reported by Valeria Orrù et al., approximately 22 million SNPs impacted 731 immune cell traits across seven groups in a study involving 3,757 Sardinian individuals. Notably, for 459 cell traits at 70 loci, the researchers identified 122 significant (*P* < 1.28 × 10^− 11^) independent association signals, with 53 being novel. Among the 731 immunophenotypes, categories included relative cell counts (*n* = 192), morphological parameters [MP] (*n* = 32), surface antigen-reflecting median fluorescence intensity (MFI) (*n* = 389), and absolute cell counts (*n* = 118) (Supplementary Table 3) [10].

### Selection of instrumental variables (IVs)

By current study guidelines, the significance level for each immunological trait’s SNPs was determined at 1 × 10^− 5^ [10, 11]. Screening only a small subset of SNPs associated with TAA occurs when the *P*-value is set to 5 × 10^-8^. To explore more plausible causal relationships and delve into the interaction between TAA and immune cells in greater detail, we adjusted the *P*-value to 5 × 10^-6^. Additionally, to mitigate the effects of strong linkage disequilibrium (LD), we employed PLINK software (version v1.90) to implement the clump method, setting the LD r^2^ threshold to < 0.1 within a distance of 1000 kb [12]. Subsequently, we calculated the F-statistic for each SNP, eliminating those with weak associations (F < 10). Finally, we utilized the screened SNPs for the two-sample MR analysis.

### Statistical analysis

For all research, R 4.3.1 (http://www.Rproject.org) was utilized.

To assess the causal association between TAA and immune cells, we employed five different methods: inverse variance weighting (IVW) [13], MR-Egger [14], weighted median [15], weighted model [16], and simple model [17]. Next, the horizontal pleiotropy was explained and ruled out using the MR-PRESSO [18] and MR-Egger approaches. Simultaneously, we assessed the differences between various instrumental variables using Cochrane’s Q statistics. Finally, a leave-one-out analysis was conducted to evaluate the impact of each SNP on MR. The results of the calculations are presented visually through a forest plot, scatter plot, and funnel plot.

## Results

### The causal effect of TAA on immunophenotypes

**Fig. 1:**
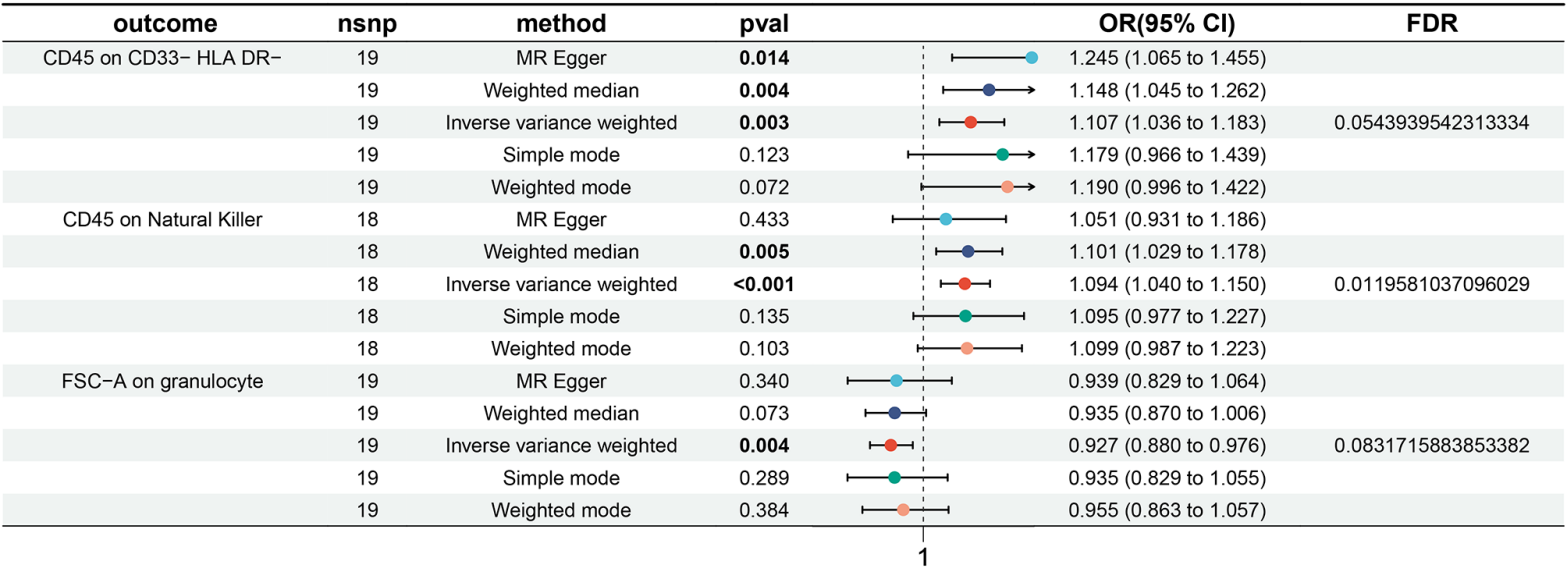
Causal Relationship between TAA and immune cell traits were showed by forest plots. TAA: thoracic aortic aneurysm

Initially, we conducted a two-sample MR analysis to examine the impact of TAA on immune traits. The False Discovery Rate (FDR) approach was employed for *P*-value adjustment, with the Inverse Variance Weighting (IVW) method serving as the primary analytical technique. A P_FDR_ of 0.05 was set, resulting in the observation of one immune trait. Subsequently, by increasing the P_FDR_ to 0.1, we identified three immune cell traits. Notably, the presence of TAA was associated with increases in CD45 on CD33^−^ HLA DR^−^ (β = 0.102, 95% CI = 1.036–1.183, *P* = 0.003, P_FDR_ = 0.054, Fig. 1, Supplementary Table 1) and CD45 on Natural Killer (β = 0.089, 95% CI = 1.040–1.150, *P* < 0.001, P_FDR_ = 0.012, Fig. 1, Supplementary Table 1), while FSC − A on granulocyte exhibited a decrease (β = -0.076, 95% CI = 0.880–0.976, *P* = 0.004, P_FDR_ = 0.083, Fig. 1, Supplementary Table 1). These associations were corroborated by four other MR techniques: MR Egger, Weighted Median, Simple Mode, and Weighted Mode (Fig. 1, Supplementary Fig. 2). The robustness of these causal associations was further assessed through Leave-One-Out analyses, evaluations for horizontal pleiotropy, and heterogeneity tests (Supplementary Fig. 1). Scatter plots and funnel plots were employed as visualizations to depict the results (Supplementary Figs. 1 & 2).

### The causal effect of immunophenotypes on TAA

**Fig. 2:**
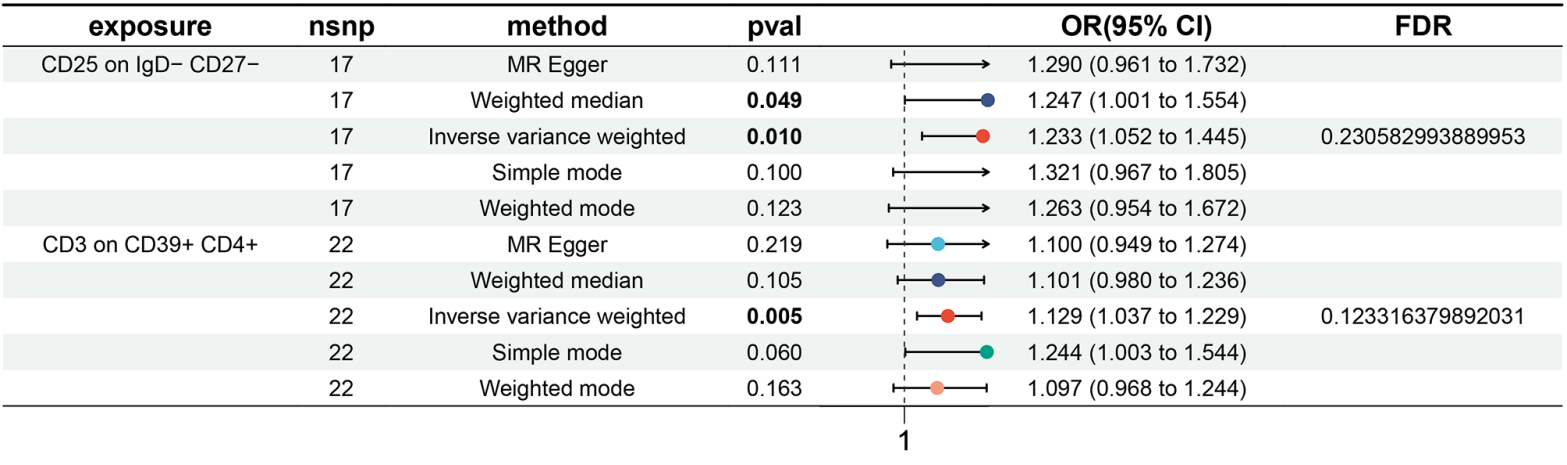
Causal Relationship between immune cell traits and TAA were showed by forest plots. TAA: thoracic aortic aneurysm

We applied the same approach to investigate the impact of immune traits on TAA. Despite being unable to identify any immunophenotype substantially correlated with TAA after FDR adjustment (P_FDR_ < 0.05), two intriguing immunological characteristics emerged when we relaxed the significance threshold to 0.30. Specifically, associations were observed on CD3 on CD39^+^ CD4^+^ (β = 0.121, 95% CI = 1.037–1.229, *P* = 0.005, P_FDR_ = 0.123, Fig. 2, Supplementary Table 2) and CD25 on IgD^−^ CD27^−^ (β = 0.209, 95% CI = 1.052–1.445, *P* = 0.010, P_FDR_ = 0.231). These connections were validated by four additional MR techniques: MR Egger, Weighted Median, Simple Mode, and Weighted Mode (Fig. 2, Supplementary Fig. 4). The strength of these causal linkages was further verified using heterogeneity tests, horizontal pleiotropy evaluations, and Leave-One-Out analysis (Supplementary Fig. 3). Visual representations, such as scatter plots and funnel plots, can be used to illustrate the results (Supplementary Figs. 3 & 4).

## Discussion

There has been substantial discourse regarding the connection between immunity and the pathophysiology of TAA. In our investigation, we explored the causal relationship between 731 immune cell traits and TAA using publicly available genetic datasets. Notably, three immunophenotypes were identified as being causally affected by TAA (P_FDR_ < 0.10), with an additional two immunophenotypes showing significant causal impact by TAA at a relaxed significance threshold (P_FDR_ < 0.30).

We observed that TAA is associated with an increase in the CD45 expression on the CD33^−^ HLA-DR^−^ subgroup of myeloid cells. However, limited information about this specific cell type is available in prior research. Our study underscores the significance of this cell type, highlighting the need for further investigation to delineate its types and functions. Additionally, we found an increase in natural killer (NK) cells following TAA. NK cells, which develop from common lymphoid progenitor cells, are large granular lymphocytes and play a crucial role in connecting adaptive immunity with innate immunity [19]. According to a study utilizing single-cell sequencing data to explore the immunogenicity of ascending thoracic aortic aneurysm, it was found that NK cells communicate with endothelial cells through the CXCL12-CXCR4 and CCL5-ACKR1 axes [20]. This intercellular communication may contribute to the development of TAA. Moreover, it is noteworthy that TAA was associated with a decrease in FSC-A on granulocytes. Within the innate immune system, granulocytes, encompassing neutrophils, eosinophils, and basophils, represent the most prevalent circulating cells. Upon activation, these myeloid polymorphonuclear leukocytes can release characteristic cytoplasmic particles into the surrounding environment [21]. Studies have revealed a rise in neutrophil infiltration within TAA [22]. Neutrophils contribute to the formation of aortic aneurysms by secreting neutrophil extracellular traps and the inflammatory molecule IL-6 [23]. While the association between TAA and eosinophils and basophils has not been thoroughly studied, we hypothesize that a decline in these two cell types may have contributed to an overall decrease in granulocyte counts.

Subsequently, we identified a significant association between the occurrence of TAA and CD3 on CD39^+^ CD4^+^ Treg and CD25 on IgD^−^ CD27^−^ B cells. Regulatory T cells (Treg), characterized by the expression of Foxp3, CD25, and CD4, exhibit notable immunosuppressive effects [24]. The reduction of the Treg cell population and impaired function in the aorta of patients with abdominal aortic aneurysms is associated with chronic inflammation [23]. Initially identified as a biomarker of activated B cells, CD39 is expressed on activated T cell subpopulations, monocytes, dendritic cells (DCs), and natural killer cells. Recent findings indicate that FOXP3^+^ Treg cells also express CD39, and ATP hydrolysis is considered a novel mechanism of Treg cell suppression [25]. Furthermore, research has revealed that Treg cells play a protective role against Aortic Aneurysms and Dissections (AAD). Depletion of Treg cells in a mouse model of AAD has been shown to lead to an increased incidence of AAD [26]. Research indicates that the predominant B cell population in experimental abdominal aortic aneurysms (AAA) is comprised of B2 cells. Moreover, in the absence of other B cell subpopulations, B2 cells have been found to suppress the development of AAA and enhance the population of regulatory T cells in the spleen [27]. These findings align with the results of our research, although further evidence will be necessary in the future.

TAA can arise from various immunological disorders, including Behcet’s disease, giant cell arteritis, big arteritis, and others. Therefore, monitoring the local and systemic inflammatory condition of the patients is beneficial for multiple purposes, such as follow-up care, perioperative management, patient stratification, TAA prevention, and surgical timing decisions. To improve patient stratification, numerous inflammatory markers have been investigated in recent decades, but their clinical translation is still pending. To establish a comprehensive monitoring system, it is crucial to explore the variations in immune cell subtypes at different stages of TAA, in conjunction with clinical features such as imaging, blood pressure, heart rate, blood lipids, and inflammation markers. This integrated approach aims to prevent aneurysm enlargement, reduce the risk of rupture, and intervene prior to the development of dissection or rupture. In addition to the common complications of rupture and bleeding, TAA can also lead to uncommon intestinal fistulas, where aortic inflammation and direct friction collaborate to spontaneously form primary fistulas [28, 29]. The infiltration of immune cells could potentially explain the structural changes in blood vessel walls. Unfortunately, the use of Mendelian randomization techniques to investigate the relationship between immune cells and aortic fistulas is not feasible due to the lack of available GWAS data for these patients.

Our investigation is subject to certain limitations. Firstly, the use of GWAS data limited to the European population resulted in incomplete data sources. To facilitate comparison with diverse ethnic origins, additional data covering a broader range of racial/ethnic groups is necessary. Secondly, the potential impact on our results from not exploring interactions between immune cells should be acknowledged. Thirdly, a larger GWAS dataset related to TAA, with an increased number of cases, is essential to ensure the screening of an adequate number of SNPs.

## Conclusion

Through our comprehensive MR investigation, we successfully mitigated the impact of potential confounding variables, reverse causal linkages, and other factors. As a result, we established a causal relationship between numerous immunological phenotypes and TAA. This discovery opens up promising avenues for further research into the roles of immune cell subtypes in multiple sclerosis, potentially providing novel insights into early intervention strategies and TAA treatments.

### Electronic supplementary material

Below is the link to the electronic supplementary material.

Supplementary Material 1

Supplementary Material 2

Supplementary Material 3

Supplementary Material 4

Supplementary Material 5

Supplementary Material 6

Supplementary Material 7

## Data Availability

The datasets used and/or analysed during the current study available from the corresponding author on reasonable request.

## Abbreviations

TAA: Thoracic aortic aneurysm
AAD: Aortic aneurysms and dissections
AAA: Abdominal aortic aneurysms
GWAS: Genome-wide association study
IVs: Instrumental variables
IVW: Inverse variance weighting
MR: Mendelian Randomization
OR: Odds ratio
LD: Linkage disequilibrium
SNPs: Single Nucleotide Polymorphisms
FDR: False Discovery Rate
NK: Natural killer
Treg: Regulatory T cells
DCs: Dendritic cells
