# Supplementary figures and images for: The causal relationship between thoracic aortic aneurysm and immune cells: a mendelian randomization study
Source: BMC Cardiovasc Disord. 2024 Apr 16;24:212. doi: 10.1186/s12872-024-03876-1 (PMC11020992; doi:10.1186/s12872-024-03876-1)

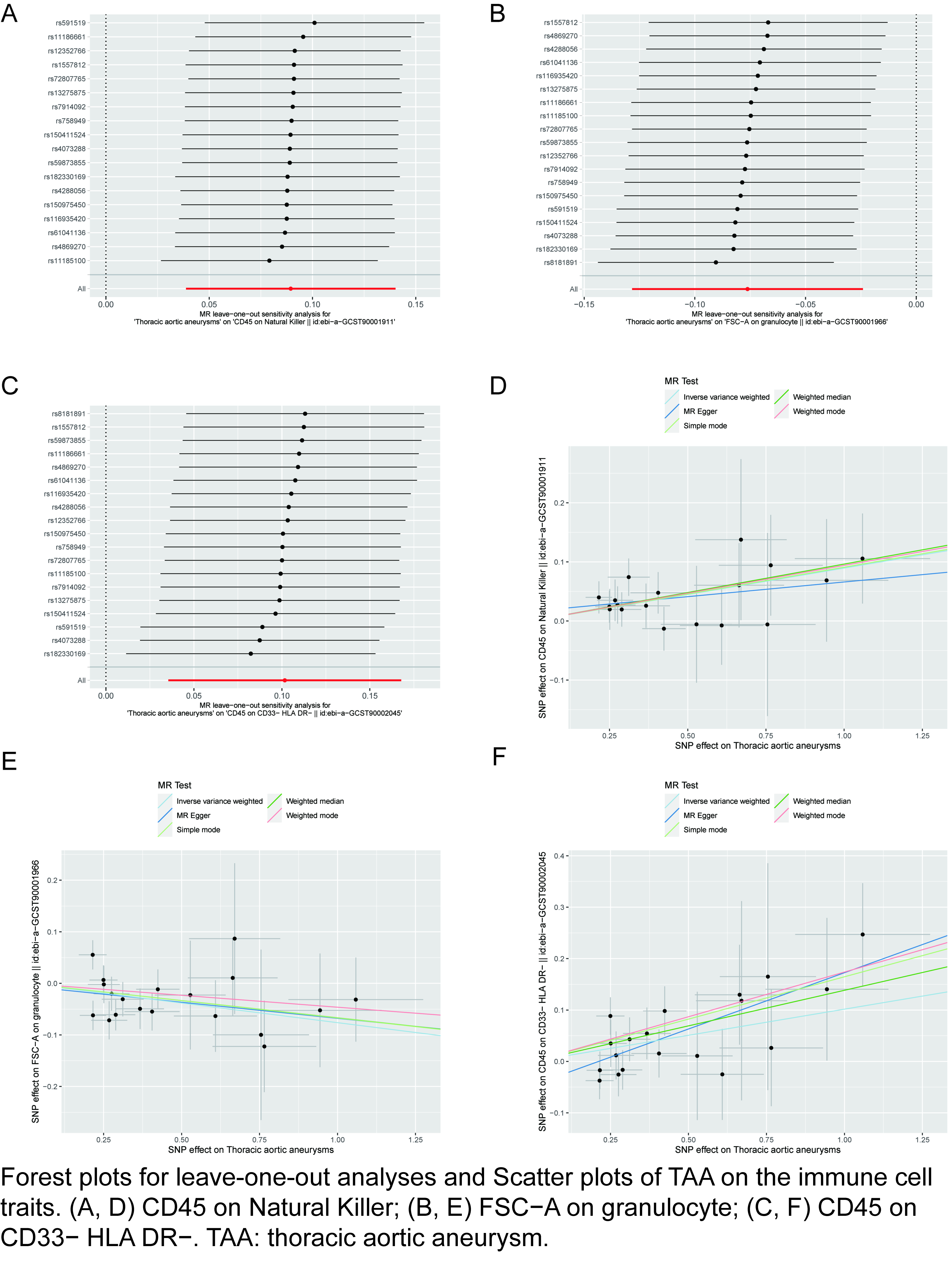

Supplement: Supplementary file 1 — Supplementary Material 1 [file 12872_2024_3876_MOESM1_ESM.tif]

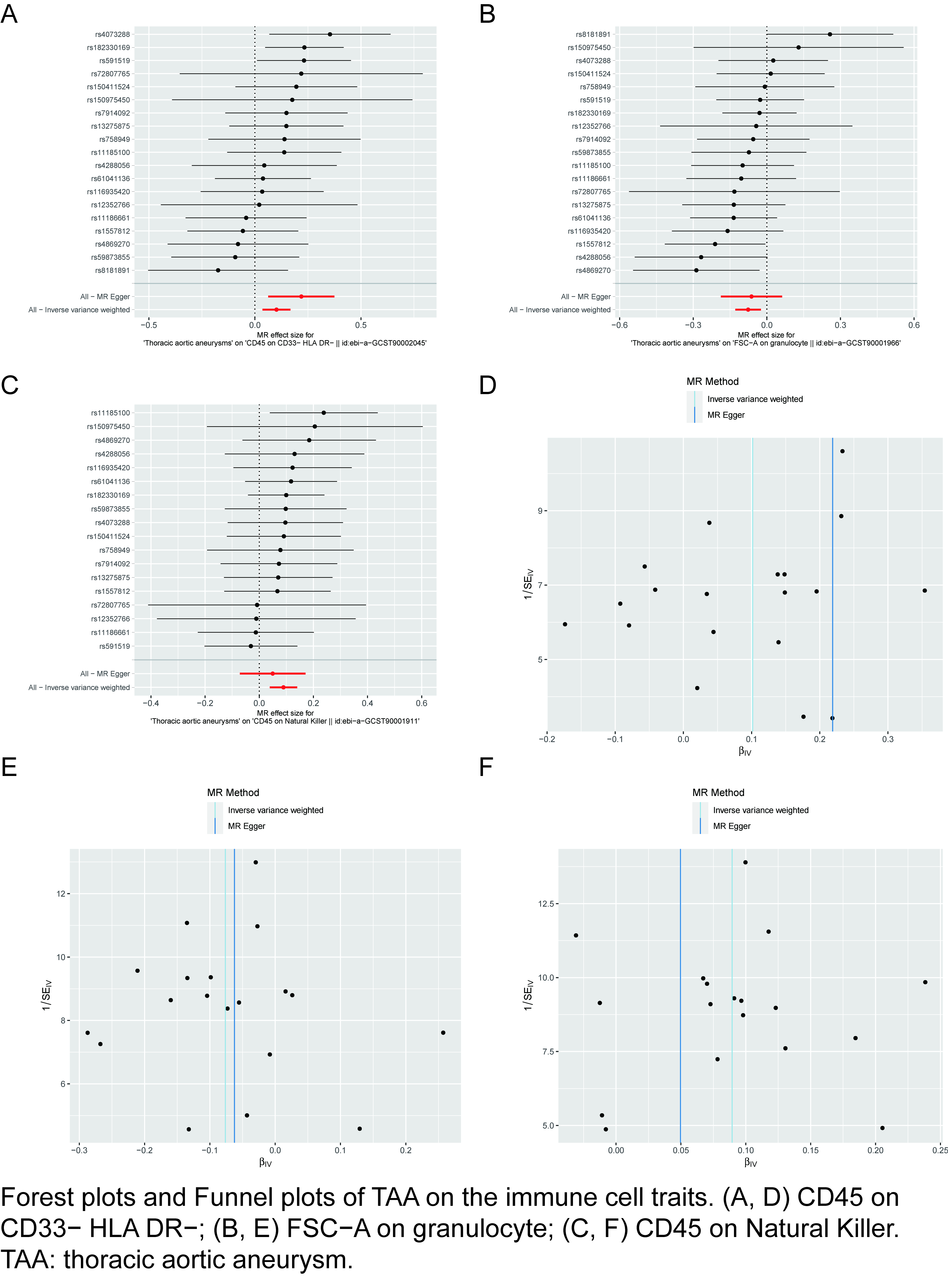

Supplement: Supplementary file 2 — Supplementary Material 2 [file 12872_2024_3876_MOESM2_ESM.tif]

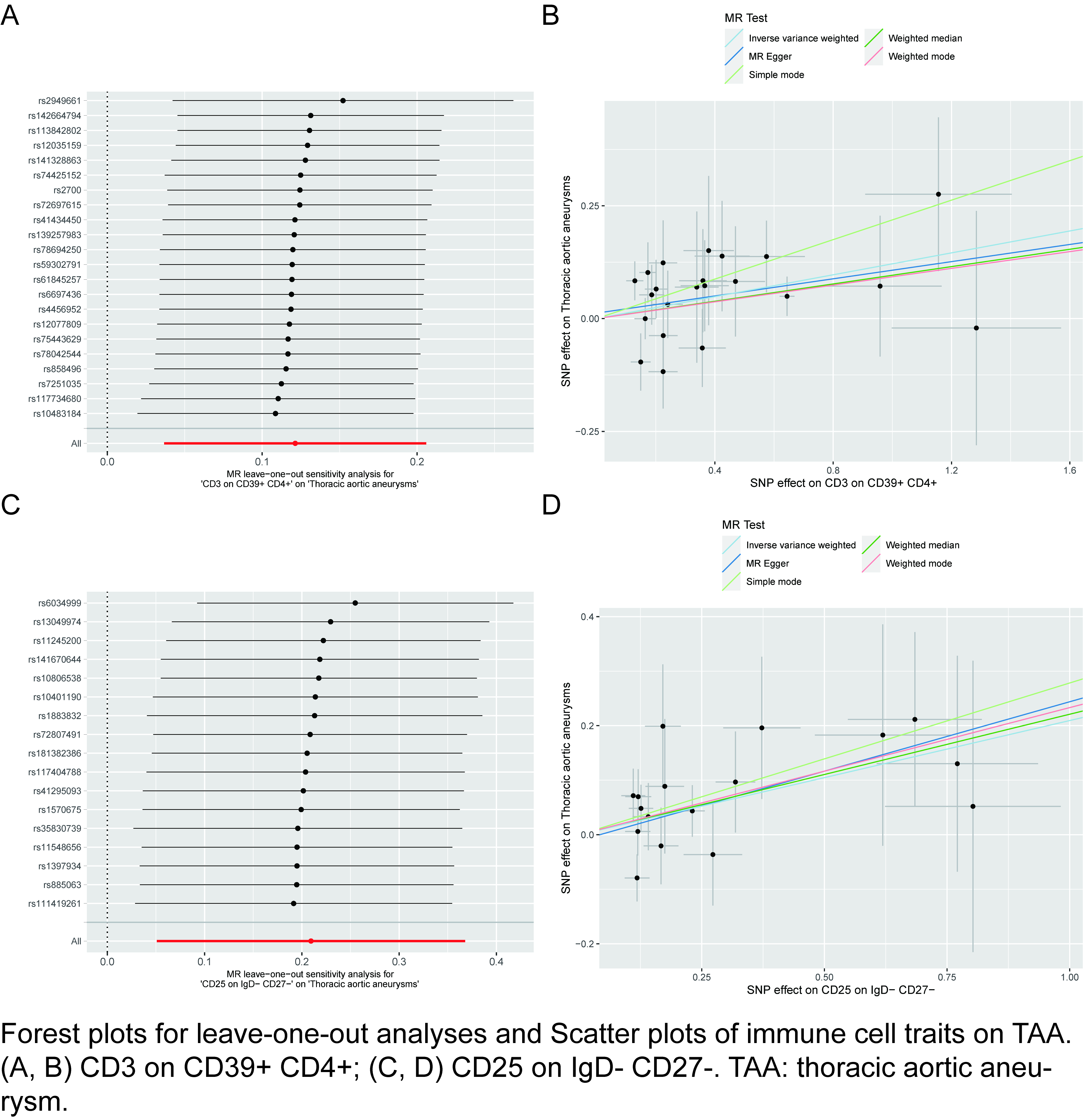

Supplement: Supplementary file 3 — Supplementary Material 3 [file 12872_2024_3876_MOESM3_ESM.tif]

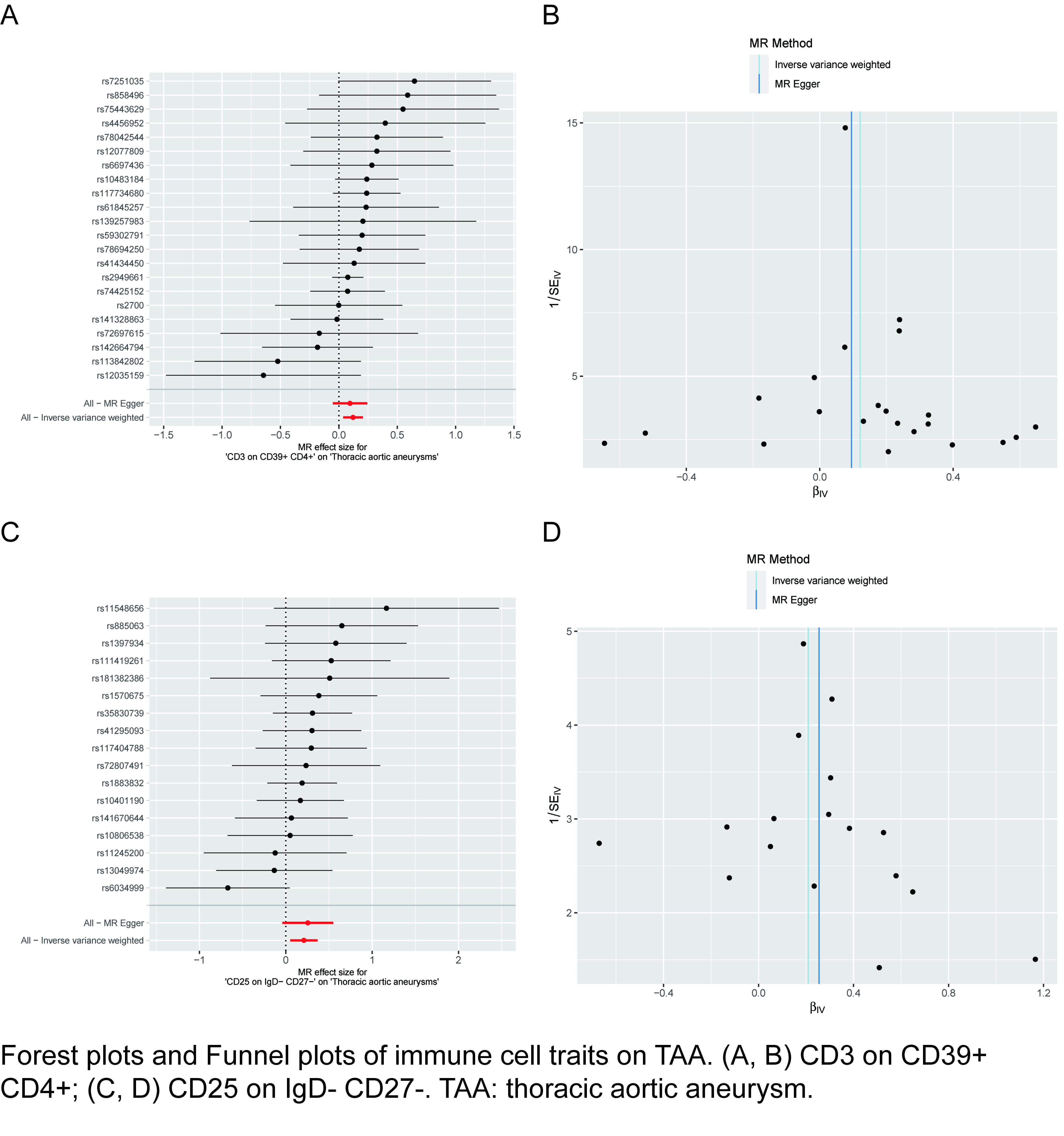

Supplement: Supplementary file 4 — Supplementary Material 4 [file 12872_2024_3876_MOESM4_ESM.tif]
